# Characterization of ultrafine particles emitted during laser-based additive manufacturing of metal parts

**DOI:** 10.1038/s41598-020-78073-z

**Published:** 2020-12-02

**Authors:** Aleksey Noskov, Torunn K. Ervik, Ilya Tsivilskiy, Albert Gilmutdinov, Yngvar Thomassen

**Affiliations:** 1 Kazan National Research Technical University, 10 Karl Marx Str., 420111 Kazan, Russia; 2 National Institute of Occupational Health, P.O. Box 8149 DEP, 0033 Oslo, Norway; 3 Institute of Ecology, National Research University Higher School of Economics, Myasnitskaya str. 20, 101000 Moscow, Russia

**Keywords:** Ecology, Health occupations, Engineering, Materials science, Mathematics and computing, Nanoscience and technology, Physics

## Abstract

Particulate matter (PM) emitted during laser additive manufacturing with stainless steel powder materials has been studied in detail. Three different additive manufacturing techniques were studied: selective laser melting, direct metal deposition and laser cladding. Gas flow and temperature fields accompanying the processes were numerically modeled for understanding particle growth and oxidation. Transmission and scanning electron microscopy were used for primary particle and PM characterization. The PM collected in the atmosphere during manufacturing consisted of complex aggregates/agglomerates with fractal-like geometries. The overwhelming number of particles formed in the three processes had equivalent projected area diameters within the 4–16 nm size range, with median sizes of 8.0, 9.4 and 11.2 nm. The primary particles were spherical in shape and consisted of oxides of the main steel alloying elements. Larger primary particles (> 30 nm) were not fully oxidized, but where characterized by a metallic core and an oxidic surface shell.

## Introduction

The processes of additive manufacturing (AM) are characterized by the incremental joining of material and have become an alternative to traditional manufacturing methods in recent years. AM is the general group of technologies that can reproduce parts of complex geometric shapes (prepared in correspondent CAD software) via successive layer-by-layer addition of material^1^. Additive equipment can operate on metals, polymers, composites, or functionally graded materials to produce structures that may be too hard or even impossible to manufacture by conventional classic technology^1–4^. Laser-based powder bed fusion of metals (PBF-LB/M) is a subset of AM whereby a heat source is used to fuse a powder material into the solid one to form three-dimensional objects^5,6^. Different ferrous and non-ferrous metallic powders as titanium and aluminum-alloys as well as iron, nickel, cobalt and copper base alloys and precious metals can be used as powder material. During the development of additive manufacturing technology there have been numerous different terms and definitions in use, often with reference to specific application areas and trademarks. This is often ambiguous and confusing which hampers communication and wider application of this technology^1^. Thus, the EOS Company has patented a process that is called as “Direct Metal Laser Sintering (DMLS)”, while the Fraunhofer Institute introduced their own term “SLM” for Selective Laser Melting. These two techniques are based on similar principles, however, to avoid any confusion we will use unified term “PBF-LB/M” according to ISO standards^2^. The Directed Energy Deposition (DED) technology based on laser treatment of powder materials and directly deposing them on the substrate, layer by layer The most commonly used materials in additive technology are metal powders or wire source materials. There are also other popular terms of DED include directed light fabrication (DLF), laser engineered net shaping (LENS), laser metal deposition (LMD), 3D laser cladding and direct metal deposition (DMD)^7,8^. Another important advantage of DED is capable to restore and heal damaged parts of complex geometric shapes, such as turbine blades or propellers^9–14^. Each method has its own characteristics and properties^14–18^. The most appropriate technique has to be chosen individually regarding the composition, precision and topology of the workpiece to be manufactured.

Under optimal conditions, high-quality work parts are obtained by AM with unique properties that cannot be obtained by traditional manufacturing processes as for example casting, forging, compression molding, milling and cutting^19–22^. However, as with all manufacturing techniques, potential occupational exposure needs to be considered. Few studies have assessed occupational exposure and characterized the airborne particulate matter emitted during 3D metal printing. Workers exposure in 12 German enterprises to dust, Fe, manganese (Mn), chromium (Cr), Co and Ni was measured by collection of both, personal and stationary respirable and inhalable air samples^23^. This study showed that the measured workroom air concentrations, especially for Ni, were higher during cleaning than manufacturing. The higher exposure during cleaning was confirmed by^24^.

It has also shown that nanometer-sized particles were present at relatively high number air concentrations in the additive manufacturing environment (Graff et al., 2016; Ljunggren, 2019)^24,25^.

Despite the fact that particulate emissions from 3D printers using different thermoplastics have been characterized (e.g., Stephens et al., 2013; Afshar-Mohajer et al., 2015; Kim et al., 2015; Azimi et al., 2016; Mendes et al., 2017)^26–30^, no information, to our best knowledge, regarding the primary particle characteristics of the nano-sized particulate matter formed during PBF-LB/M manufacturing is available. A fundamental prerequisite for evaluating potential adverse health effects among workers after inhalation of ultrafine particulate matter is the knowledge of relevant physical and chemical characteristics of the particles such as primary particle size distribution, agglomeration/aggregation state, shape and chemical composition (e.g., Oberdörster et al., 2005)^31^.

In the present contribution, ultrafine particulate matter (PM) emitted during laser processing of stainless steel powder materials is characterized in detail by transmission and scanning electron microscopy (TEM/SEM). The primary particle size, chemical and phase composition as well as mixing state of the particles are studied.

## Experimental

### Laser-Based additive manufacturing

Three industrial machines of AM have been investigated: PBF-LB/M machine by EOS M270 instrument (EOS GmbH, Krailling, Germany); DED machine by an InssTek MX-Mini instrument (InssTek, Yuseong-gu, Daejeon, Republic of Korea), and laser cladding (LC) using a LC-10 IPG-Photonics (IPG Photonics, Oxford, MA, USA) attached to a KUKA KR 120 R 2700 extra HA industrial robot (KUKA ROBOTER GmbH, Gersthofen, Germany). The experimental parameters used are summarized in Table 1. Images of the instruments can be found in the electronic supplement (Figures S1).

**Table 1:** Experimental conditions of additive laser processing.

Instrument	EOS M 270 dual mode	InssTek MX-Mini	LC-10 IPG-photonics
Technique	PBF-LB/M	DED	LC
Laser	Yb fiber laser	Yb fiber laser	Yb fiber laser
Laser power (W)	200	200–1000	500–700
Scan speed (mm s^−1^)	800	14.1	15
Layer thickness (µm)	30	250	200–250
Hatch distance (µm)	100	400	1000
T build up plate (°C)	80	28	28
Shielding gas	N_2_ (99.99%)	Ar (99.99%)	Ar (99.99%)
Gas flow rate (L min^−1^)	30	15	17

The chemical composition of the powders used for different AM techniques as measured in this work and given by the producers (values in brackets) is shown in Table 2.

**Table 2:** Chemical composition (wt. %) of powders used for additive manufacturing.

Element	Powder (additive technique)		
	EOS PH1^a^ (EOS M 270 dual mode)	Surfit 316L^a^ (InssTek MX-Mini)	AP-FeCr12MoV^a^ (LC-10 IPG-photonics)
Fe	Base	Base	Base
Cr	15 (14.0–15.5)	17 (16.0–18.0)	12.1 (11.0–13.0)
Ni	4.2 (3.5–5.5)	12 (12.0–14.0)	0.4 (< 0.4)
Cu	3.8 (2.5–4.5)	0.1	0.04
Mn	0.1 (< 1.0)	1.5 (< 0.2)	0.2 (< 0.4)
Si	0.1 (< 1.0)	0.3 (< 1.0)	0.8 (0.8)
Mo	0.03 (< 0.5)	2.5 (2.0–3.0)	1.4 (0.5–1.2)

^a^EOS M 270 dual mode: EOS PH1 stainless steel, EOS GmbH-Electro Optical Systems, Krailling, Germany; MX-Mini: stainless steel 316L-5520, Lot 2303574, 70 µm, (Höganäs Belgium S.A., Ath, Belgium); LC-10 IPG-Photonics: stainless steel AP-FeCr12MoV, JSC Polema, Tula, Russia.

### Air sampling

Bulk PM and individual particles for microscopy were collected by use of 25 mm “total dust ” “open-face” aerosol cassettes (M000025A0, Millipore, Bedford, MA, USA) operated at 2.0 L min^-1^ air flow rate using in-house produced PS103 personal pumps (National Institute of Occupational Health, Oslo, Norway). The cassettes were equipped with 25-mm polyvinyl chloride (PVC) filters with 5 µm pore size (Merck Millipore, Burlington, MA, USA).The same cassettes were additionally used to collect particles for electron microscopy. Copper TEM grids with holey carbon films (EMresolution, Sheffield, UK) were affixed to the surface of the PVC filters used as substrates.

An air pump equipped with filter cassette was placed in each of the two ventilation outlets at both sides of the EOS 270 M270 cabinet while four pumps with cassettes were placed inside along the cabinet walls of InssTek MX-Mini with each filter cassette facing about 25 cm from and 20 cm above to the laser spot. During operation of the LC-10 IPG-Photonics equipment two cassettes were mounted at a cast iron support stand with clamps facing with a distance of about 20 cm from and 20 cm above to the laser spot. Air sampling times varied between 20 and 20 min for InssTek MX-Mini and LC-10 IPG-Photonics and between 2 and 7 min for EOS 270.

**Figure 1:**
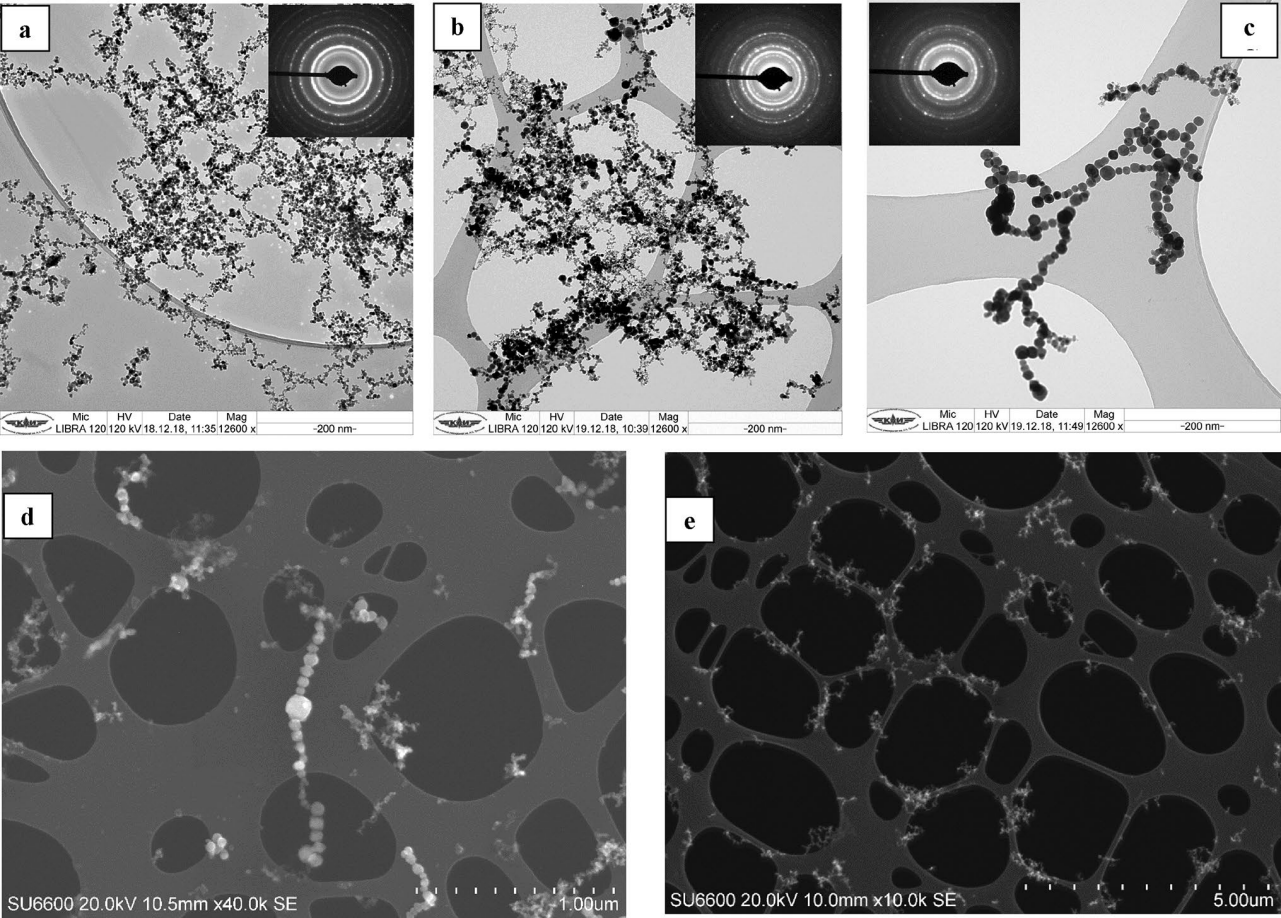
TEM Bright-field images and selected area electron diffraction patterns (inserts) of aggregates/agglomerates encountered in additive laser processing of stainless steel powder: **(a)** EOS M 270 dual mode, **(b)** InssTek MX-Mini, **(c)** LC-10 IPG-Photonics with an aggregate/agglomerate with large primary particles, **(d)** LC-10 IPG-Photonics: a SEM image with both small and large primary particles and **(e)** EOS M 270 dual mode: a SEM overview image of deposited agglomerates.

The deposition pattern of the PM on the TEM grids is visualized in SEM images, Fig. 1c,d.

In case of overloading of PM during sampling this would mainly have influenced the possibility to characterize the size distribution of the generated aggregates/agglomerates. However, since the objective of this work was to characterize the primary particles formed, an overload of PM on the grids would cause negligible effects. However, the two SEM micrographs indicate that the collected individual particles were deposited without any significant interference of overload.

### Elemental composition of powders

The elemental composition of the three stainless steel powders used was measured after 10 mg portions were dissolved in a mixture of 2 mL of aqua regia and 0.5 mL of hydrofluoric acid with microwave-assisted heating (MLS 1200, Teflon container SV140, 10 bar, Milestone, 556 J. Sorisole, Italy). Before digestion, 10 µg of beryllium (as chloride) was added as internal standard before dilution to 15 mL with de-ionized water. The elemental composition was measured with a Perkin-Elmer Optima Model 7300 DV inductively coupled plasma optical emission spectrometer (Perkin-Elmer Inc., Waltham, MA, USA).

After removing the TEM grids from the air filter surface, the same analytical procedure was used for measurement of the elemental content of the particulate matter collected by the filters.

### Scanning and transmission electron microscopy

The TEM grid specimens were imaged and analyzed in a Hitachi SU6600 field emission scanning electron microscope (Hitachi, Tokyo, Japan) equipped with a Bruker energy-dispersive X-ray detector (Bruker Nano GmbH, Berlin, Germany) and a NORDIF electron backscatter (EBSD) detector (NORDIF, Trondheim, Norway). For elemental analysis of particles, an acceleration voltage of 15 keV, analytical working distance of 10 mm and electron probe current 7–8 nA were used. For aggregates/agglomerates with primary particle sizes less than 20 nm, an area of approximately 100 × 100 nm was scanned for X-ray acquisition in a particle–dense area to obtain elemental spectra. For larger particles (> 30 nm) X-ray spectrums from single particles were obtained. In addition, an Auriga Crossbeam Workstation (Carl Zeiss AG, Oberkochen, Germany), equipped with INCA X-Max silicon drift detector (Oxford Instruments, Abingdon, UK) for energy dispersive X-ray microanalysis was used.

The phase and elemental composition of the particles were studied by a Zeiss Libra 120 transmission electron microscope equipped with an OMEGA energy filter (Carl Zeiss AG, Oberkochen, Germany). Particle diameter measurements were conducted by statistical analysis of TEM images using the Minitab version 16 software (Minitab Statistical Software, Minitab 16; https://www.minitab.com).

### Numerical modelling

The ANSYS FLUENT CFD software (ANSYS Inc., Canonsburg, PA, USA, https://www.ansys.com) was used for mathematical modelling of gas-phase processes that mimics operating conditions of the EOSINT-M270 dual mode and the InssTekMX Mini techniques. Computational domain of 2 mm width consists of two regions for solid (of 0.35 mm in height) and gas phase (of 1 mm in height). Solid phase is Steel 316, and gas is nitrogen. Ambient conditions correspond to typical working atmosphere in the chamber with 101,325 Pa pressure and 310 ° K temperature. Laser heating is emulated via volumetric heat source sliding along with the surface of solid phase with a velocity of 0.7 m/s. Laser spot radius equals R = 0.05 mm and its penetration depth is D = 0.1 mm. Using powder absorbance of A = 0.7 (for infrared radiation source) and laser power of P = 100 W, one can compute volumetric dissipated power of that heating source as Q = A × P / (π × R^2^ × D) = 89,127 GW/m^3^. Under such conditions the steel within the keyhole will immediately evaporate forcing significant convective upstream right above the heat affected zone covered by the laser spot. Metals vaporize mostly from a thin layer of few mean free paths of molecules above the heated surface, so called Knudsen layer. Recoil velocity of hot vapour can be estimated ^34^ by the expression: *U*_*vap*_ = (γ × *k *× *T*_*vap*_/*m*)^(1/2)^, where γ = 1.67 is adiabatic constant, *k* is the Botlzmann constant, *m* = 56 is molar mass in a.m.u, and *T*_*vap*_ = 3400 K is a metal boiling point. Using this formula, the vapour velocity is estimated to about 930 m/s which was added in FLUENT via the user-defined-function interface as a custom spot-wide momentum source (in the Navier–Stokes equation) moving with the laser scanning speed.

## Results and discussion

Atmospheric cabinet concentrations of elements emitted as ultrafine PM during additive laser processing are given in Table 3.

**Table 3:** Atmospheric concentrations of elements (mg/m^3^) measured during laser additive processing (n = 2).

Element	EOS M 270 dual mode	InssTek MX-Mini	LC-10 IPG-Photonics
Fe	0.98	0.60	0.13
Cr	0.24	0.27	0.02
Ni	< 0.040	0.14	< 0.015
Mn	< 0.007	0.37	< 0.005

### Primary particle formation and size-distribution

**Figure 2:**
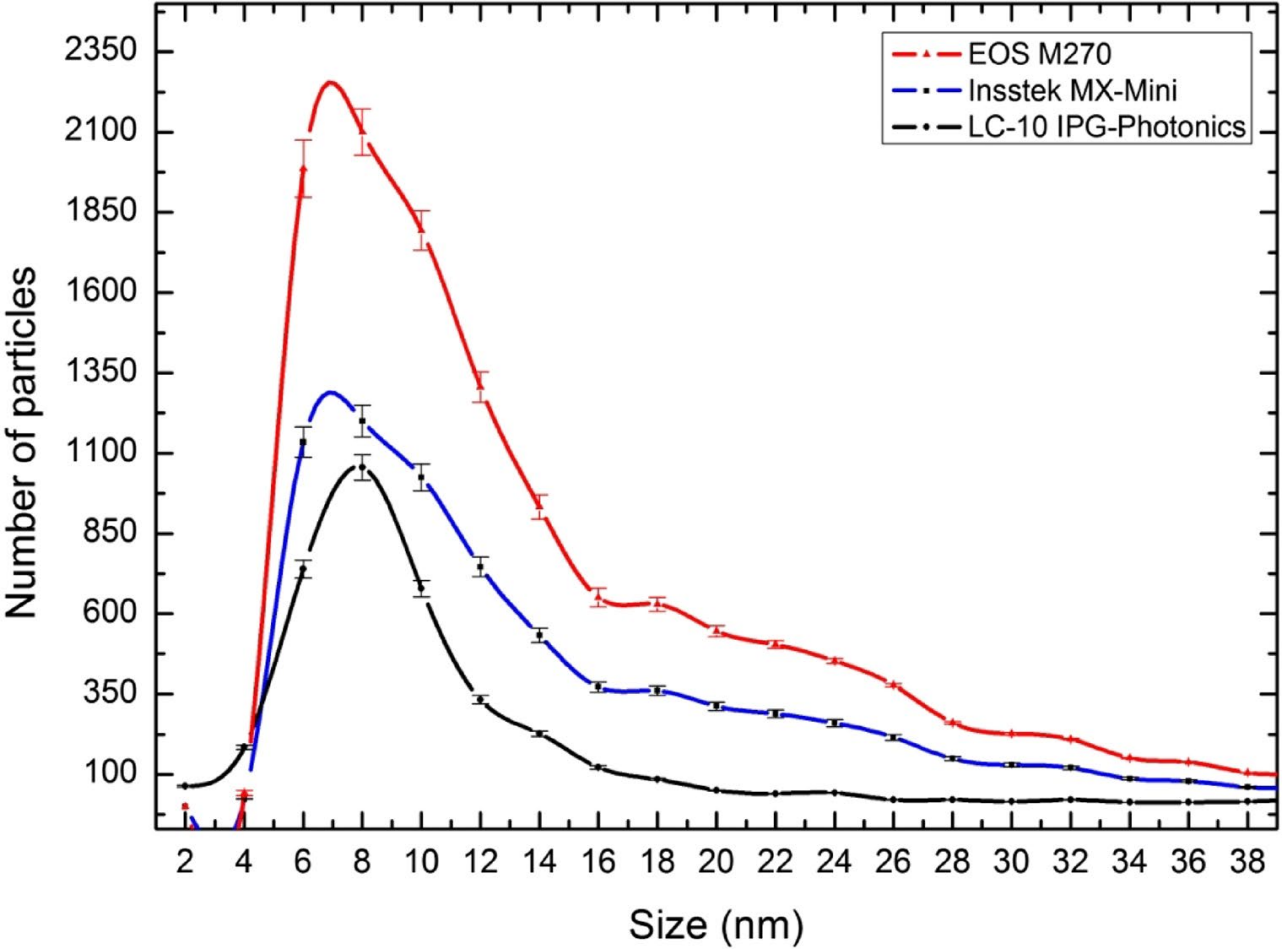
Size distribution (equivalent projected area diameter) of primary particles. Calculated by Minitab 16 software (Minitab Statistical Software, Minitab 16; https://www.minitab.com).

For all three instruments studied, the collected PM consisted of complex aggregates/agglomerates with fractal-like geometry (Fig. 1). No more than ten coarser particles with geometric projected diameters between 0.7 and 2 µm were observed on each filter. The elemental compositions were similar to the bulk material and no crystalline phases were identified. The presence of these may be due to sputtering from the melted alloy. No larger particles were seen. An equivalent projected area diameter of primary particles measured by TEM are shown in Fig. 2 and primary particle size-distribution summary statistics is presented in Table 4. The overwhelming number of particles formed in the three processes had equivalent projected area diameters within the 4–16 nm size range, with median sizes of 8.0, 9.4 and 11.2 nm for EOS M 270 dual mode, InssTek MX-Mini and LC-10 IPG-Photonics, respectively. The largest primary particles identified in the size-measurements had diameters of 50.4, 82.0 and 77.5 nm, correspondingly. Compared to previous research of laser ablation of metals where a maximum of the particle size distribution at 6–11 nm, dependent on laser intensity, were observed, the sizes of the primary particles in the laser additive processes studied in this work are similar^32^. It has previously been shown that the PM generated during manual metal arc, metal inert gas (MIG) and tungsten inert gas welding operations consists of agglomerates with primary particle diameters in the range of 5–40 nm with very few above 50 nm^33^.

**Table 4:** Summary statistics of primary particle sizes in nm.

Machine	EOS M 270 dual mode	InssTek MX-Mini	LC-10 IPG-Photonics
Minimum	2.1	3.9	1.1
Lower quartile	6.1	7.3	6.1
Median	9.4	11.2	8.0
Upper quartile	14.2	18.8	11.8
Maximum	50.4	82.0	77.5
N^a^	8494	6312	3020

^a^Number of primary particles investigated.

### Numerical modelling of gas-phase processes

**Figure 3:**
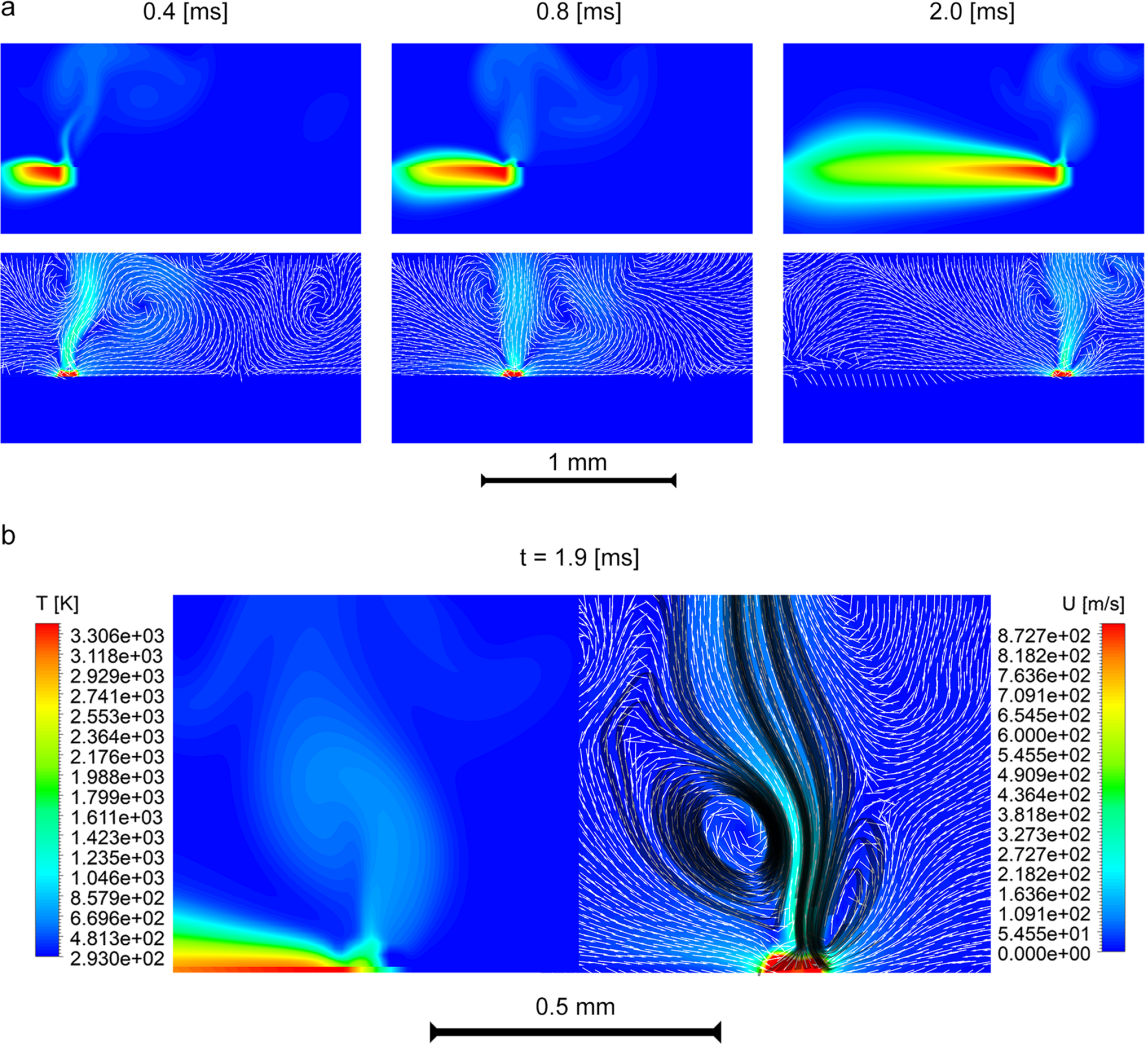
Simulated temperature and velocity fields during laser processing using EOS M270 dual mode. **(a)** Dynamics of process. Top: temperature, bottom: velocity magnitude and normalized vectors. Color mapping is the same as is shown in part **(b)** of this figure. Laser moves from left to the right side. **(b)** Temperature, velocity and streamlines (in black) close to keyhole. Computations and post-processing have been performed in Ansys Academic Research Fluent, Release 19.2 https://www.ansys.com/products/fluids/ansys-fluent.

To understand particle growth and oxidation it is essential to locate their trajectories in the zones of heated laser spots. Although it is difficult to visualize tracks of nano-sized particles directly because of their size, as well as gas flow dynamics in vicinity of processed zone, it is, however, possible to perform a close-to-real-life numerical simulation of these gas-phase processes during laser surface treatment of the substrate. In Fig. 3 the presence of toroidal eddies surrounding a hot vertical jet of metal vapour is demonstrated. These vortices remain unchanged in vicinity of a laser spot during all the process of sintering and form a recirculation zone around the heat-affected region. A close look with streamlines plotted from a base of this hot up-stream (Fig. 3b) shows the recirculation zone more clearly. Nano-sized particles due to their extremely low mass (about 5 × 10^–16^ ng) will exactly follow gas streamlines, finally trapping into that toroidal eddies. However, the particles do not stay in the recirculation zone permanently: they grow and drift to peripheral regions of the vortex, and finally leave it. According to estimations based on our numerical simulation, particle mean residence time in a vortex is about 0.5 ms for a particle of initial diameter of 10 nm and density of 7850 kg/m^3^.

**Figure 4:**
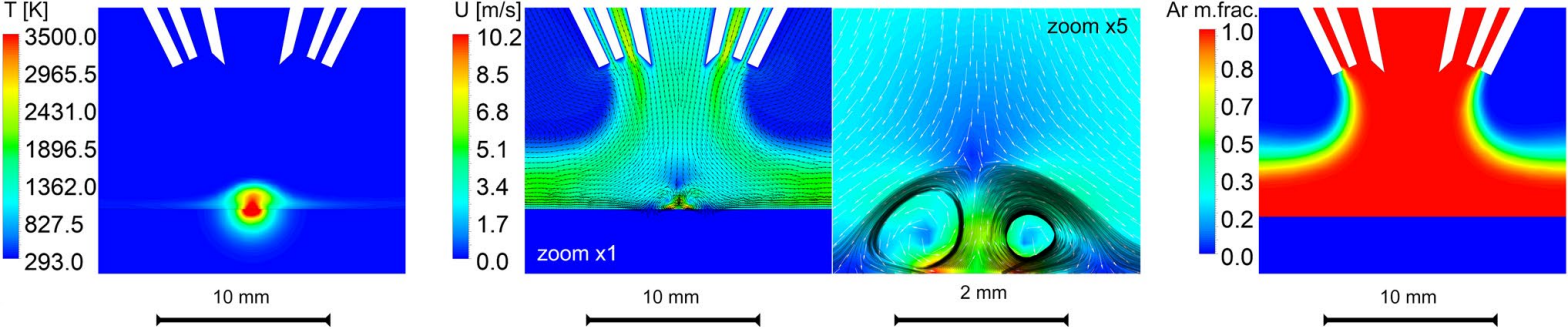
Simulation of InssTek MX Mini: temperature, velocity and mass fraction of argon. Velocity vector field and streamlines are zoomed in vicinity of a heat-affected zone. Computations and post-processing have been performed in Ansys Academic Research Fluent, Release 19.2 https://www.ansys.com/products/fluids/ansys-fluent.

Similar behavior of particles is expected for the DED machine. To verify that, gas flow and temperature dynamics for the InssTek MX Mini machine have also been simulated. Laser power of 200 W is focused in a Gaussian beam having a spot diameter of 1 mm, whereas surface absorbance and penetration depth are the same as in the modeling of the PBF-LB/M machine. Although complete information on inner design of a nozzle mounted in that machine is not available, we primarily oriented on its general view and typical conventional flow rates used in three-stream coaxial nozzles. Computational domain is initially filled with air, and all in-nozzle inlets consist of 99.9% pure argon. We consider even this “approximate” case is still usable to estimate flow pattern near the laser spot. Although the cladding head moves horizontally with speed of 1 cm/s, the flow field is relatively steady. Vaporization-induced puff above the treated surface of steel has a height of about 1 mm. Again, streamlines sampled close to the heat-affected zone represent the gas recirculation regions which are shown in black color on top of the vector velocity field, demonstrating the occurrence of a toroidal vortex caused by the hot vertical gas stream (Fig. 4). In contrary to the PBF-LB/M process, this gas jet cross-collides with a flow moving in opposite direction produced by the cooling gas. Nano-particles (NP) of partially condensed metal vapor should thus be trapped and turned back to the laser-affected zone again, but some of them will follow peripheral streamlines and slide along the treated surface. Estimated particle in-eddy residence time approximately equals 1.5 ms which is about 3 times more than in the PBF-LB/M process.

The boundary zone of the vortex is located close to the mixing zone of the surrounding air (see Ar mass fraction in its mixture with air in the right part of Fig. 4, where “Ar-air” interface is marked in green color) with a likely forced oxidation of airborne particles because of their possible interaction with oxygen (O) from air.

Various fabrication methods of NP based on vapor deposition have been developed. Laser ablation is a method where very high energy is focused to a solid material for evaporation of light-absorbing materials where the vapor phase is thermodynamically unstable. Under chemical supersaturation vapor-phase atoms/molecules will rapidly and uncontrolled be condensed with a coagulation rate proportional to the square of their number concentration. At high temperatures particles coalesce faster than they coagulate; at lower temperatures loose agglomerates with rather open structures are formed^35^. In high temperature aerosol reactors NP (< 5 nm) coalesce almost takes place instantaneously even at temperatures considerably lower than the melting temperature of the bulk material due to e.g. the reduced melting temperature of NP^36^. The primary particle growth is also dependent on the metal vapour concentration. It has been shown in a simulation of fume formation mechanism in arc welding that particles are mainly generated by FeO nucleation with small sizes formed at low Fe concentrations/low temperatures and larger primary particles at higher Fe concentrations which are not fully oxidized because of their lower surface to volume ratio^37^.When aggregates/agglomerates are allowed to be exposed to high temperatures the whole or part of the particle may be restructured during sintering even until a fully coalesced sphere is formed^36^. Similar particle formation mechanisms in PBF-LB/M processes are expected (Fig. 1b,c) where both restructured and fully coalesced spheres are present in particles consisting of both loosely and sintered primary particles. In our simulation of the gas-phase processes it is shown that when metal powder is rapidly heated by the laser, a mushroom-like cloud of hot metal vapour is formed just above the laser-processing zone of the metal surface. The evaporation rate in PBF-LB/M is more intensive than in DED because of smaller laser spot diameters (providing higher dissipated power density) used in PBF-LB/M. When released from the metal surface, the vapor rapidly expands into the surrounding atmosphere where fast-moving gas jets with surrounding toroidal eddies are formed with a typical speed of 930 m/s.

These vortices remain unchanged in the vicinity of the laser spot forming a recirculation zone around the heat-affected zone. Thus, the vapor will rapidly be transported to relatively cold regions with following condensation. In the case of DED, condensed low-mass particles follow the streamlines of the toroidal vortices located on the boundary of vapor cloud. This boundary surface is located remarkably close to the argon-air interface allowing further low-temperature oxidation of particles and their subsequent growth. Once particle diameter reaches its critical value (due to continuous cooling and oxidation), the particle may release from these vortices because of inertia forces and gas flow instabilities with abortion of further growth.

Evolution of processes in gas metal arc welding fume has been investigated by Vishnyakov et al.^38,39^ by numerical modeling. According to their model it was shown that the primary particle chemical composition and the particle size distribution strongly depends on the vapor–gas mixture cooling rate. Such a dependency was explained in their model by the decrease of the vapor–gas mixture cooling rate when the shielding gas temperature was increased. Therefore, duration of particles growth via vapor condensation and coalescence is increased with subsequent increase of particle size. The number based primary particle size distributions presented by^38^ compare well with our results of particle size-distributions during additive processing (Table 4 and Fig. 2). This may indicate that the mechanism of formation and growth of primary particles during AM is similar to the processes occurring during arc welding.

Analysis of gas flow in a cross-section of the domain shows that there are two primary gas jets affecting the flow pattern: carrier gas-powder stream and shielding gas protecting the optics. It looks like increased flow rates of shielding gas (simultaneously maintaining the carrier gas flow unchanged) will lead to efficient removal of airborne particles out the zone of processing preventing their remelting in a circulated toroidal vortex of the carrier gas. Higher flow rates will also possibly lead to decrease of particle oxidation due to their reduced residence time in the air-argon mixture on the boundary of nozzle-produced gas streams and ambient atmosphere. This is a typical scenario of gas-flow-particle interaction. However, to establish exact relation between gas flow rates and powder removal rate a series of additional numerical simulations under various operating conditions will be done in further.

### Elemental composition of primary particles

**Figure 5:**
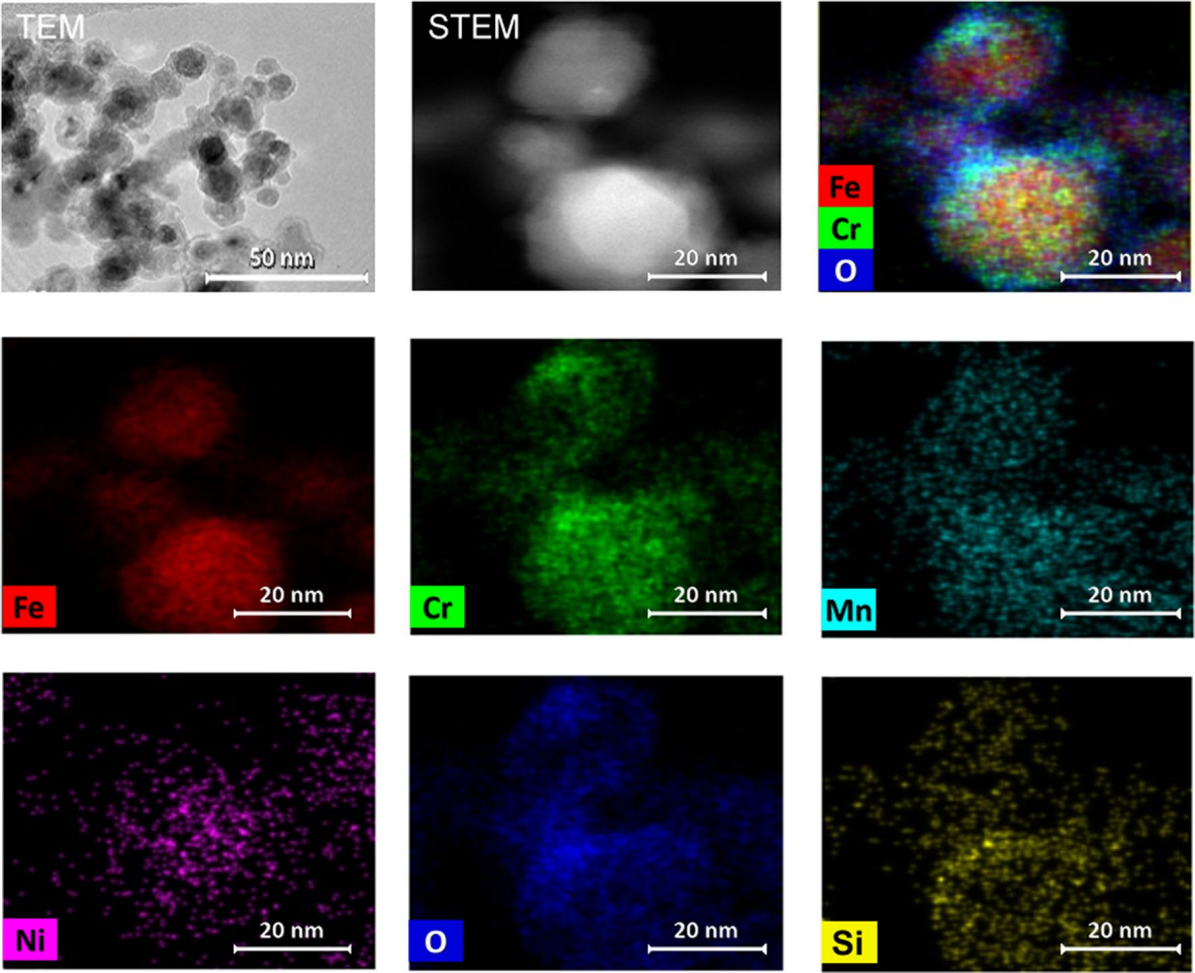
TEM bright field image, high-angle annular dark-field STEM image and element distribution images of aggregates from additive laser processing with EOS M 270 dual mode.

**Figure 6:**
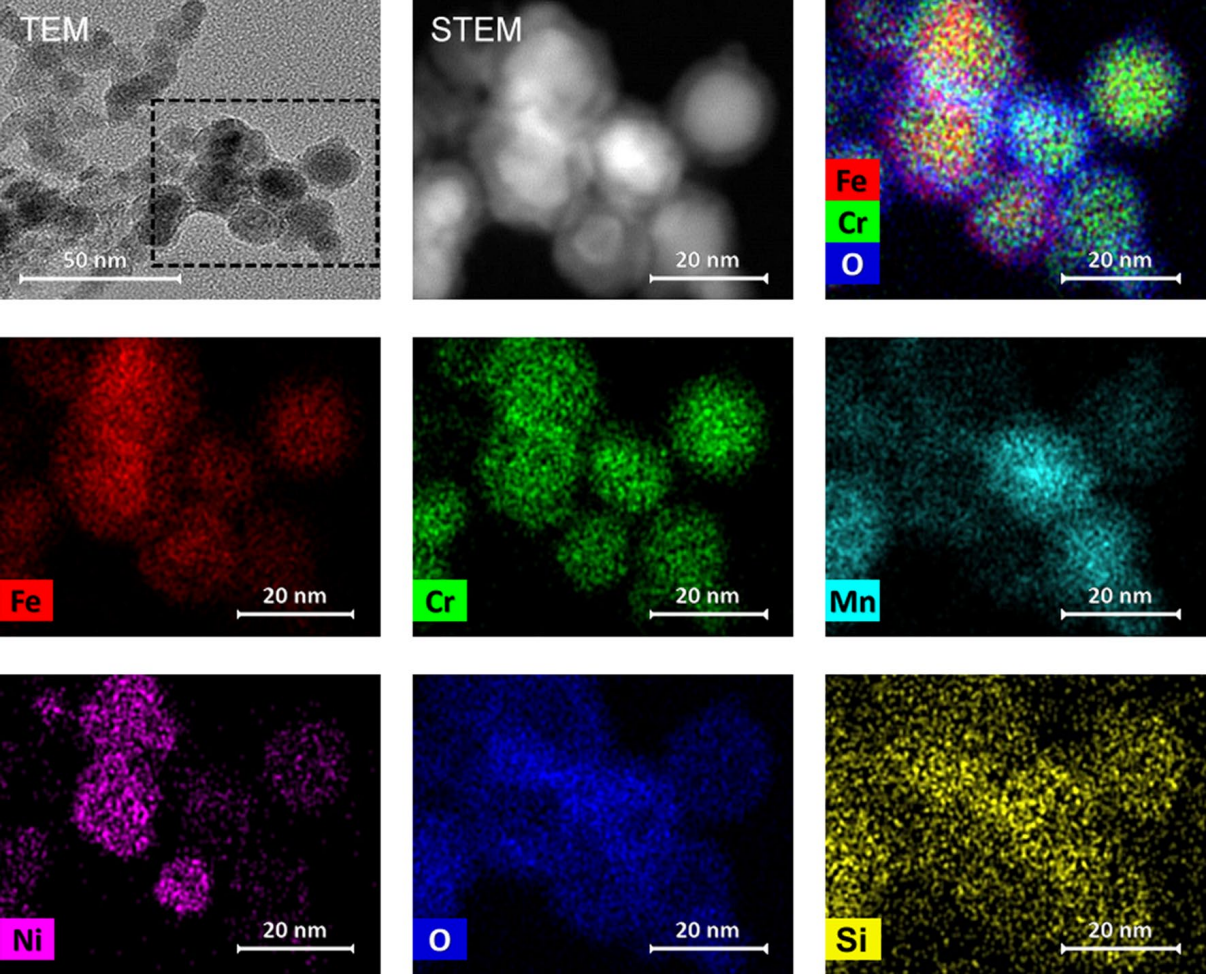
TEM bright field image, high-angle annular dark-field STEM image and element distribution images of aggregates from additive laser processing with InssTek MX-Mini.

**Figure 7:**
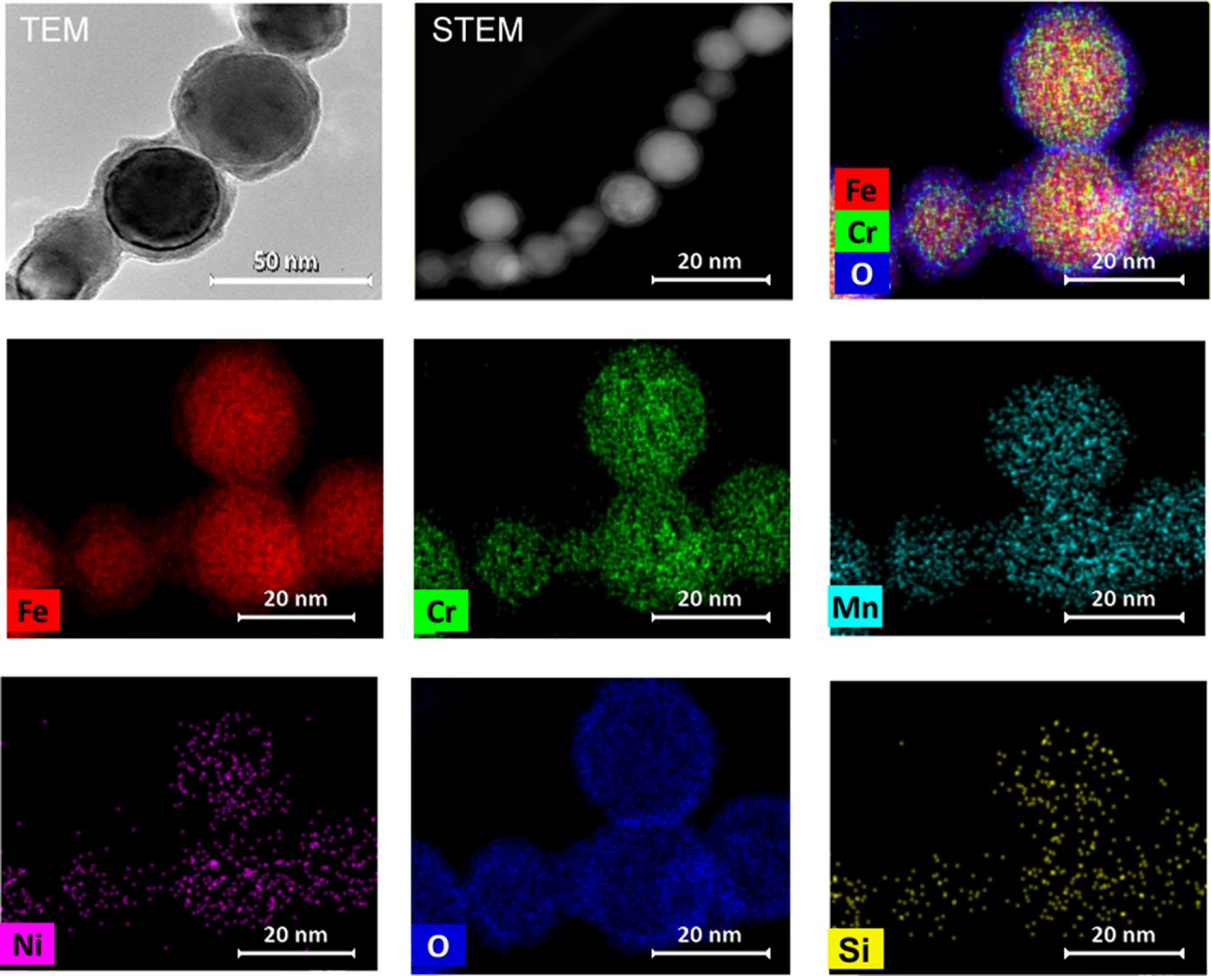
TEM bright field image, high-angle annular dark-field STEM image and element distribution images of aggregates from additive laser processing with LC-10 IPG-Photonics.

At high magnification (Figs. 5, 6, 7) it is noticeably that the primary particles are sintered to each other leading to aggregates which are held together by chemical and/or sinter forces^40,41^. The primary particles in the present size range are usually spheres with a core shell structure (Figs. 5, 6, 7). In these samples the 20 nm sized particles predominantly consist of the main alloying elements Fe, Cr and Ni in addition to Mn, Si and O which are all more or less homogeneously distributed in the particles as shown in the elemental maps in Figs. 5 and 6 for EOS M 270 and InssTekMx-Mini, respectively. For the 30–50 nm sized particles generated during operation of the LC-101PG-Photonics machine, there is indications of less O in the core, but an O enriched shell around the particles as illustrated in Fig. 7.

**Figure 8:**
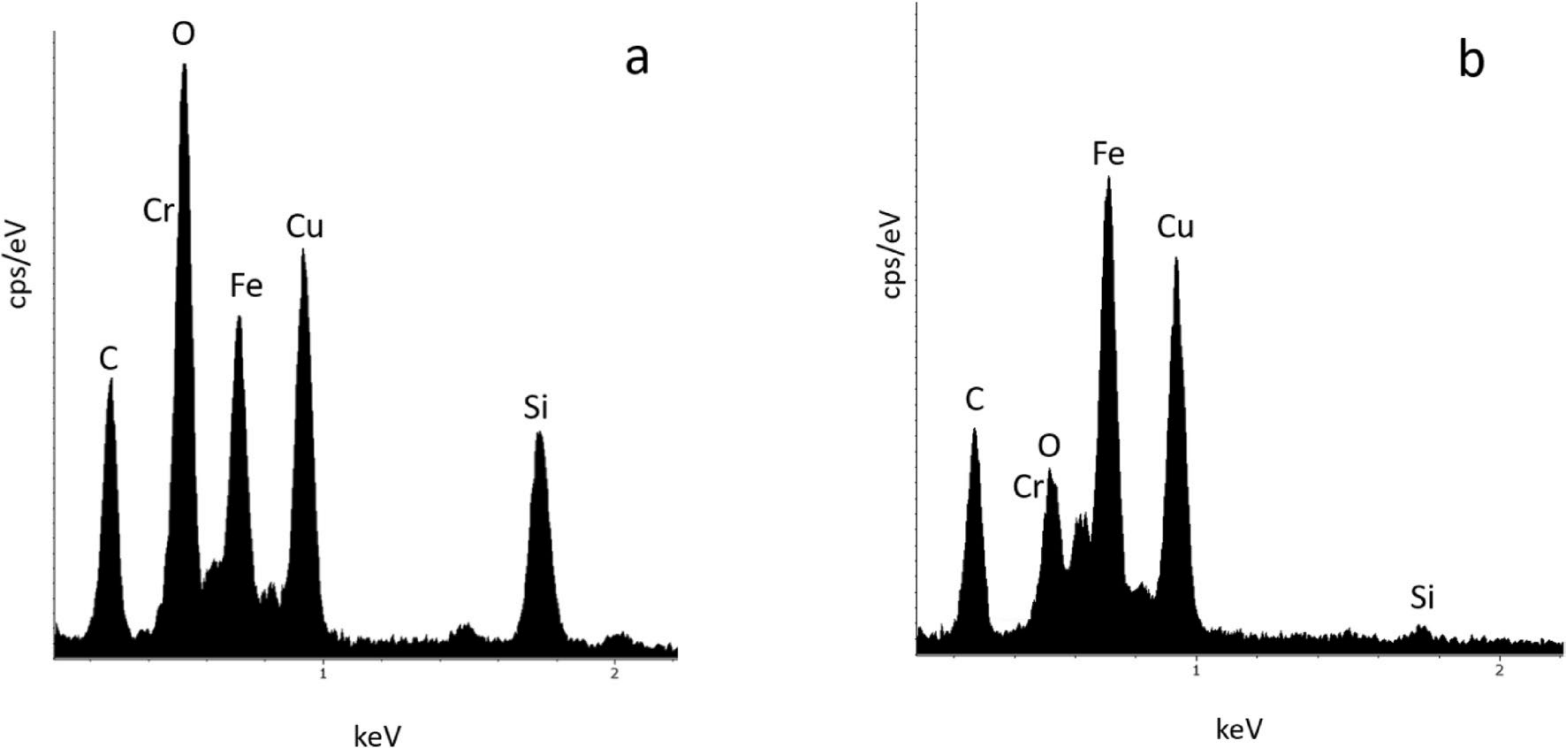
EDX spectra of **(a)** aggregates with primary particles < 20 nm and **(b)** individual 50 nm primary particles from additive laser processing with LC-10 IPG-Photonics.

Since it was not possible to quantitatively measure the elemental composition of the individual primary particles due to their small size (< 20 nm) the major element content of the aggregates was determined by scanning the electron beam over an area of approximately 100 nm × 100 nm with roughly the same number of primary particles (Fig. 8, Table 5). In addition, the larger particles (30–50 nm) from LC-10 IPG-Photonics were analyzed. Aluminum, carbon, copper and tin which were detected in all samples were excluded, as they are artifacts from the TEM grids and the substrate, respectively. Since the EDS X-ray analysis system has a default spectra for the elements, the measurement differences between these spectra used for estimating the elemental composition and our TEM grid samples, limits the possibility to obtain quantitative data because appropriate ZAF correction factors could not be obtained. Especially for O, this is critical^42^. The amount of Fe and Cr in PM, (shown as Fe/Cr mass ratios in Table 5) quantified from SEM spectra of agglomerates and analysis of air filters is comparable to the content in stainless steel alloys used in the experiments. The ratios for Ni and Mn indicate fractionation of both elements with increased content in PM for the InssTek MX-Mini process. Especially for Mn, the fractionation is significant. For the other methods, valid elemental ratios were not obtained due to absence of usable quantitative information in the measurements.

**Table 5:** Mass ratios between Fe, Cr, Ni and Mn in particulate matter formed during additive laser processing of stainless steel alloys.

Machine	Fe/Cr			Fe/Ni		Fe/Mn	
	Alloy	SEM	Air filters	Alloy	Air filters	Alloy	Air filters
EOSM 270	5.1	4.1^a^	4.1^c^				
InssTek MX-Mini	3.9	4.4^b^	2.3^c^	5.4	4.3^c^	43	1.6^c^
LC-10 IPG	7.0	21^a^	6.8^c^				

^a^n = 20.

^b^n = 15.

^c^n = 5.

By modeling Fe fume formation in arc welding^37^ it has been shown that Fe atoms react in the vapour phase with O to form vaporous Fe monoxide (FeO) at 2500 K followed by nucleation of the FeO to liquid particles at temperatures around 2000–2500 K; remaining Fe atoms are condensing on the particle surface. Further on, the liquid particles are oxidized to Fe_3_O_4_ around 2000 K with solidification around 1800 K. Fe is further oxidized to Fe_2_O_3_ around 1500 K, but this is only for the smallest primary particles due to diffusion limitations. Thus, at the end of the formation process, the composition of the primary particles depends also on the initial Fe and O amounts. Larger primary particles are not fully oxidized since their surface to volume ratio is lower which prevent O diffusion into the core of the particles. The low O content relative to Fe present in large particles (approx. 50 nm) from LC-10 IPG shown in Fig. 7 may be explained by Sandibondi’s model^37^.

Selected area electron diffraction (Fig. 1a,b,c) reveals that the PM consist of crystalline phases. Furthermore, the particles were investigated with EBSD in SEM which earlier has been used to characterize phase compositions of particles^41^. The EBSD patterns, shown in Fig. S2 and S3, are a Fe_3_O_4_ spinel phase for the EOS M 270 dual mode and α-Fe for the LC-10 IPG-Photonics. EBSD is a single particle analysis principle and it might very well be that a mixture of the two phases are present for all three techniques. Because of the small size of the primary particles, it is expected that the EDSD signals originate from the particle core. The shell surrounding the particles are clearly an oxide layer and could be Fe_3_O_4_. These results support also the formation process suggested by Sanibondi’s model^37^.

### Health aspects

Inhalation is the most relevant exposure route for occupational exposure to ultrafine PM. The lung deposition characteristics, the potential toxic effect induced and the kinetic fate and possible translocation to other organs are predominantly mostly determined by the agglomeration/aggregation status of the inhaled ultrafine PM^42^. Scheckman and McMurry^43^ have shown experimentally using a silicone rubber lung cast model that silica agglomerates with primary particle diameter of 10 nm deposited more efficiently than sodium chloride (NaCl) particles and oleic acid (OA) spheres with equal mobility and aerodynamic sizes in the size ranges 30–300 nm. At larger primary particle sizes the deposition pattern for the agglomerates was closer to match that of NaCl and OA. This may indicate that the ultrafine PM characterized in the present work, if inhaled, would deposit more efficient than predicted using the International Commission on Radiological Protection and/or multiple-path particle dosimetry models^44,45^.

If it is considered that deposited agglomerates/aggregates consist of about 10 nm sized primary particles as characterized in the present study, that are more or less loosely bound to each other, the crucial question for assessing possible biological effects of these particles upon inhalation is, if the agglomerates remain as agglomerates or if the agglomerates break down to smaller aggregates or even primary particles in contact with the lung surface—which may significantly influence their toxicological properties^46^.

Buckley et al.^47^ exposed rats nose-only to aerosols of radioactive iridium-192 particles with sizes ranging from 10 to 75 nm demonstrating a slow lung clearance and increasing concentrations of particles with decreasing particle size in secondary target organs as liver and kidney with a translocation efficiency of max 0.5% of the lung burden. If this low dose build-up of particles in other organs than lung could lead to any systemic effects is still unclear in their opinion.

Animal experimental studies, in vitro and in vivo, have demonstrated the tendency of nano-sized particles to form larger size agglomerates following deposition and an increase of particle number due to disintegration of agglomerates seems not to be of high relevance^42^.

Therefore, particle sizes measured airborne in the respiratory zone of individuals seem to be a reasonable estimate of the size related properties of particles in the lungs.

To our best knowledge the primary spherical particles emitted in this work with a core composition of mainly Fe, Cr, Ni and Mn and an oxidic coated surface have not been toxicology tested. However, comparable primary particles in the same size range and chemical composition are generated during solid stainless steel wire welding^48^. When such particles have been investigated in lung cells and reporter cell lines, they showed no toxic effects to the reporter cells, no cytotoxicity, genotoxicity and no generation of reactive oxygen species^49^.

## Conclusion

Our study shows that considerable amounts of ultrafine particles are emitted during the laser additive processing of stainless steel powder material. From high-resolution electron microscopy characterization and numerical modelling it has been shown that the PM emitted in the three additive processing technologies consists of aggregates/agglomerate with primary equivalent projected area diameters predominantly within the 4–16 nm size range and with median sizes of 8.0, 9.4 and 11.2 nm. The primary particles were spherical in shape and consisted of oxides of the main steel alloying elements. Larger primary particles (> 30 nm) were not fully oxidized, but where characterized by a metallic core and an oxidic surface shell. According to the simulation of the laser processing of metal powder, it can be assumed that the particles aggregate close to the thermal heating zone, where secondary melting of ultrafine particles also take place. Detailed examination of primary particles indicated that they are sintered to each other leading to aggregates where particles are bound together by chemical and/or sinter forces. The primary particles are usually oxide spheres with a pronounced core shell structure. All particles contained the main elements present in the stainless steel alloy processing powder used.

Comparable PM with primary particles in the same size range and chemical composition are generated during solid stainless steel wire welding.

## Supplementary information

Supplementary Figures.
