# Supplementary material for: Characterization of ultrafine particles emitted during laser-based additive manufacturing of metal parts
Source: Sci Rep. 2020 Dec 2;10:20989. doi: 10.1038/s41598-020-78073-z (PMC7710759; doi:10.1038/s41598-020-78073-z)
Supplement: Supplementary file 1 — Supplementary Figures. [file 41598_2020_78073_MOESM1_ESM.docx]

| a | b | c |
| --- | --- | --- |
| 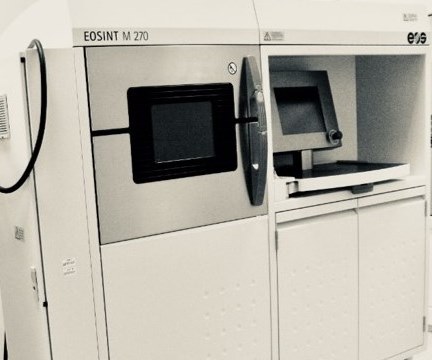 | 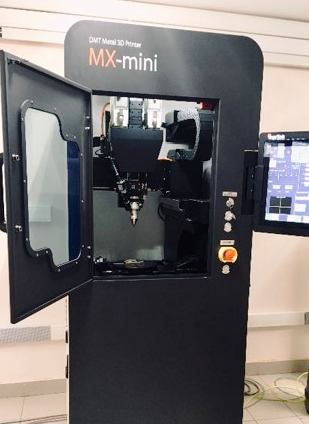 | 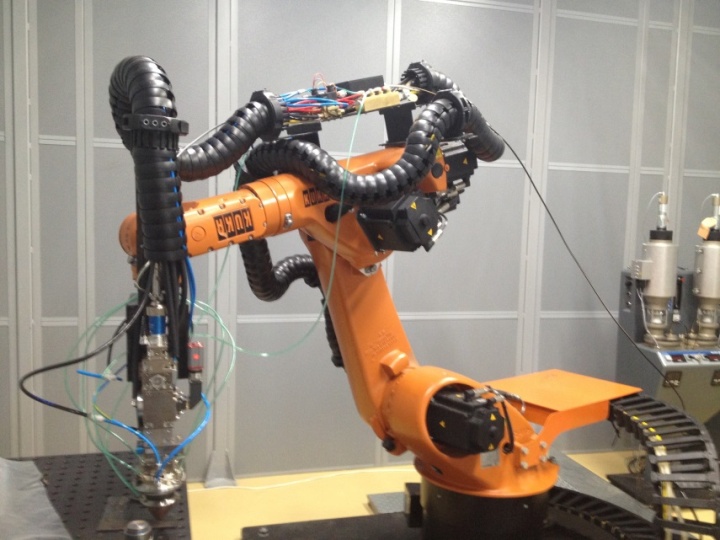 |

**Figure S1:**a**)** EOS M 270 dual mode; b**)** InssTek MX-Mini instrument; c**)** LC-10 IPG-Photonics laser cladding system

a b


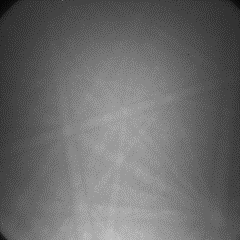

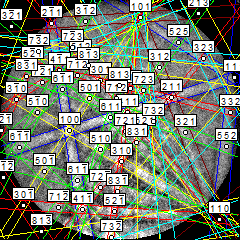


**Figure S2:** a**)** Raw EBSD pattern from particles from additive laser processing with EOS M 270 dual mode. b) indexed EBSD pattern shows magnetite (Fe_3_O_4_).

a b


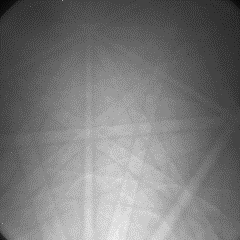

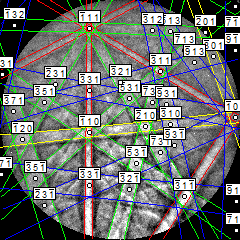


**Figure S3: a)** Raw EBSD pattern from a particles from additive laser processing with LC-10 IPG-Photonics. b) indexed EBSD pattern shows α-Fe.
